# Global high-resolution growth projections dataset for rooftop area consistent with the shared socioeconomic pathways, 2020–2050

**DOI:** 10.1038/s41597-024-03378-x

**Published:** 2024-05-30

**Authors:** Siddharth Joshi, Behnam Zakeri, Shivika Mittal, Alessio Mastrucci, Paul Holloway, Volker Krey, Priyadarshi Ramprasad Shukla, Brian O’Gallachoir, James Glynn

**Affiliations:** 1 SFI MaREI Centre for Energy Climate and Marine, Cork, Ireland; 2 Environmental Research Institute, University College Cork, Cork, Ireland; 3 School of Engineering, University College Cork, Cork, Ireland; 4 Energy, Climate, and Environment Program, International Institute for Applied Systems Analysis (IIASA), Laxenburg, Austria; 5 Institute for Data, Energy, and Sustainability (IDEaS), Department of Information Systems and Operations Management, Vienna University of Economics and Business (WU), Vienna, Austria; 6 Grantham Institute – Climate Change and the Environment, Imperial College London, London, UK; 7 CICERO Center for International Climate Research, Oslo, Norway; 8 Department of Geography, University College Cork, Cork, Ireland; 9 Industrial Ecology Programme and Energy Transitions Initiative, Norwegian University of Science and Technology (NTNU), Trondheim, Norway; 10 Global Centre for Environment and Energy, Ahmedabad University, Ahmedabad, India; 11 Center on Global Energy Policy, Columbia University, New York, USA; 12 Energy Systems Modelling Analytics, Galway, Ireland

**Keywords:** Energy policy, Sustainability, Climate-change mitigation, Socioeconomic scenarios, Climate-change policy

## Abstract

Assessment of current and future growth in the global rooftop area is important for understanding and planning for a robust and sustainable decentralised energy system. These estimates are also important for urban planning studies and designing sustainable cities thereby forwarding the ethos of the Sustainable Development Goals 7 (clean energy), 11 (sustainable cities), 13 (climate action) and 15 (life on land). Here, we develop a machine learning framework that trains on big data containing ~700 million open-source building footprints, global land cover, road, and population datasets to generate globally harmonised estimates of growth in rooftop area for five different future growth narratives covered by Shared Socioeconomic Pathways. The dataset provides estimates for ~3.5 million fishnet tiles of 1/8 degree spatial resolution with data on gross rooftop area for five growth narratives covering years 2020–2050 in decadal time steps. This single harmonised global dataset can be used for climate change, energy transition, biodiversity, urban planning, and disaster risk management studies covering continental to conurbation geospatial levels.

## Background & Summary

Global building stock consumed circa 18% of the global electricity demand and contributed to 21% of the global GHG emissions in the year 2019^1^. United Nations^2^ projects that the global population will grow from 8 billion in 2022 to 9.7 billion by 2050. The increase in population will require an increase in global building stocks and will have increasing downstream effects on material demands^3^. In contemporary literature, rooftop areas or in general vector building footprints with additional enrichment of building types, floor area per capita, construction year etc. are often used as a reliable proxy for generalising global building stock^4^.

Hence, a harmonised global geospatial assessment of global rooftop area assessment is essential for various research domains, including urban planning and architecture^5^, renewable energy^6^, and sustainable development^7^ as it provides crucial data for optimising space usage, designing sustainable buildings, fostering renewable energy adoption, and improving the overall environmental performance of urban areas. The availability of a harmonised dataset that documents the global rooftop area is of importance to not only energy system modellers but also to national and international research institutions as this spatially explicit dataset can aid in energy planning, access to energy, analysing impacts of extreme natural events^8^ and conflicts^9^. Of more importance is that a first order harmonised spatially explicit dataset be generated that documents the future spatial growth in the rooftop area to aid in cross-domain scenario analysis and policy formulation by incorporating different socioeconomic growth dynamics to fulfil the complementary needs of Sustainable Development Goals and mitigation of climate change.

Global assessment of gross rooftop area is a complex task as the smallest unit of assessment is a rooftop. This complexity is compounded by the fact that building stock archetypes change between geographies and are dependent on the socio-economic and cultural factors prevalent in the region of interest (ROI). In the past, bottom-up modelling approaches^10–14^ were used to assess the rooftop area at sub-national and national scales. Here, the studies focussed on the extrapolation of relationships between socioeconomic drivers and rooftop areas from a small sample region to a larger ROI. Although these methods are useful for rapid estimation of rooftop areas, they often report lower accuracies than the highly spatially resolved methods that utilise large-scale surveying of building stocks^15^.

On the other hand, highly spatially resolved top-down^16–20^ techniques like Light Detection and Ranging (LiDAR) based rooftop mapping which use a drone-mounted laser to map the landscape in 3D,and Machine Learning (ML) based object detection have shown promising results for ROIs covering continental scales. The LiDAR-based rooftop mapping is currently the most accurate method of determining the rooftop area along with capturing the rooftop attributes at scale. But these methods require significant investment in aerial imaging and computational costs because of which the most common implementation of LiDAR-based rooftop mapping is limited to a city scale analysis. ML-based models form the next class of methods that can aid in the detection of building rooftops at scale. However, these methods have shown limited suitability for a global scale study as the training of ML models requires heavy investment in training data that should have enough diversity to cover a global ROI^21^. Additionally, a server-scale computational environment is required to train and generate inferences from these trained ML models which requires significant cost and time investment. As a result of this, the largest ROI tackled by an ML-based approach covers the continent of Africa^20^. However, extending this to a global implementation is yet to be achieved due to complexities around capturing accurate geographically diverse samples to train the ML models and the prohibitive cost of mapping the globe using LiDAR. Moreover, the application of the top-down method has been restricted to a single-year estimation of rooftop area and only limited studies have researched into advancing the bottom-up methods to future high-resolution estimation of growth in global rooftop area^22^.

A third stream of methods that can aid in the rapid assessment of rooftop areas at ROIs spanning continental scales is to use a hybrid approach. This approach utilises the spatial relationship among samples covering landcover mapping (derived from remotely sensed imagery), socioeconomic metrics and actual on-ground building stock attributes to infer rooftop areas for out-of-sample regions. Studies that have demonstrated this hybrid approach^18,23^ utilise statistical inferencing to generate these relationships for Continental and country-level ROI.

**Fig. 1:**
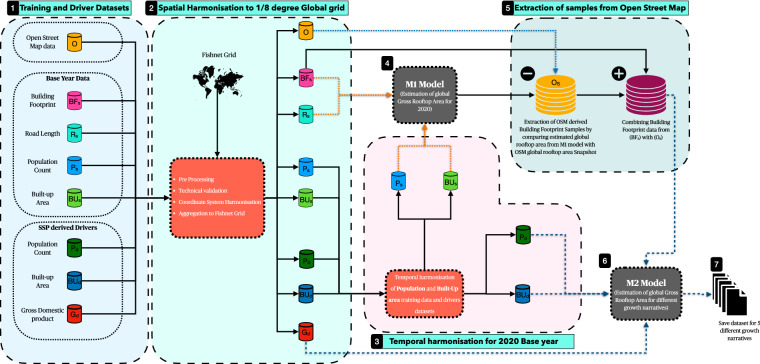
Flowchart illustrating the methodology of this study. The workflow was executed in seven steps (marked in back boxes). The workflow started with the collection of training and driver datasets, followed by spatial and temporal harmonisation of collected datasets. Next, we generated rooftop area estimation for the year 2020 in step 4 which was further used to select samples from Open Street Map datasets. In the sixth and seventh steps, we generated estimates of growth in the global gross rooftop area.

For this study, we combined the bottom-up and top-down approaches to develop a hybrid ML-based framework built on our previous learnings from a single-year global estimation of rooftop solar PV^6^. The hybrid ML framework learns from the spatial relationship between downscaled Gross Domestic Product (GDP)^24^, Population density^25,26^, built-up area extent^27^, and sample building footprints to estimate rooftop area in out-of-sample regions. The Shared Socioeconomic Pathways (SSP) narratives^28^ which are extensively used in climate change research, examine how global society, demographics and economics might change over the next century by quantifying the narratives into numerical metrics that can be interpreted by mathematical models. The framework for SSPs starts with a narrative defining five different worlds based on challenges to adaptation and mitigation. SSP1 is the sustainable world, SSP3 is the world under regional rivalry having the highest challenges to mitigation and adaptation, SSP4 is the world of inequality with the highest challenge to adaptation, SSP5 is the fossil-fuelled world with the highest challenge to mitigation and, SSP2 is the middle of the road pathway. By using SSP-specific spatially explicit growth in GDP^24^, population density^29^, and build-up area^30^ as drivers to the trained ML framework, we estimated the growth in the global building footprint area which we one-to-one map as gross rooftop area under each of these development pathways, Fig. 1. This way we combine the spatial attributes (built-up area) of top-down modelling with statistical modelling (socioeconomic parameters) of bottom-up methods. The hybrid ML framework allows for estimating the global gross rooftop area by leveraging the global statistical relationship between sample building footprint, built-up area on-ground, population and GDP which mitigates the need for an extensive ML-based building polygon extraction from remotely sensed images while providing accuracies in the range of ±0.1 km^2^ in predicted rooftop area per 1/8-degree fishnet grid tile. Another advantage of the hybrid ML framework over top-down ML-based approaches is the low computational footprint of the framework which precludes the use of image processing and hence reduces the barrier to access for open-source big data like building footprints, global road datasets etc.

## Methods

### Data collection

We started the task of data collection by defining a global fishnet (FN) grid at a spatial resolution of 1/8 degree. The FN grid cell has an approximate spatial resolution of 14 km^2^ at the equator and the size of the grid cell is dynamic based on the latitude it lies in but maintains the same 1/8-degree length and height. This spatial resolution of the grid was chosen to match the spatial resolution of the SSP-derived population and built-up extent gridded datasets. A 14 km^2^ FN grid resolution provides us with a large enough extent to capture city limits at scale and a small enough extent to not cover the entire conurbations within itself.

**Fig. 2:**
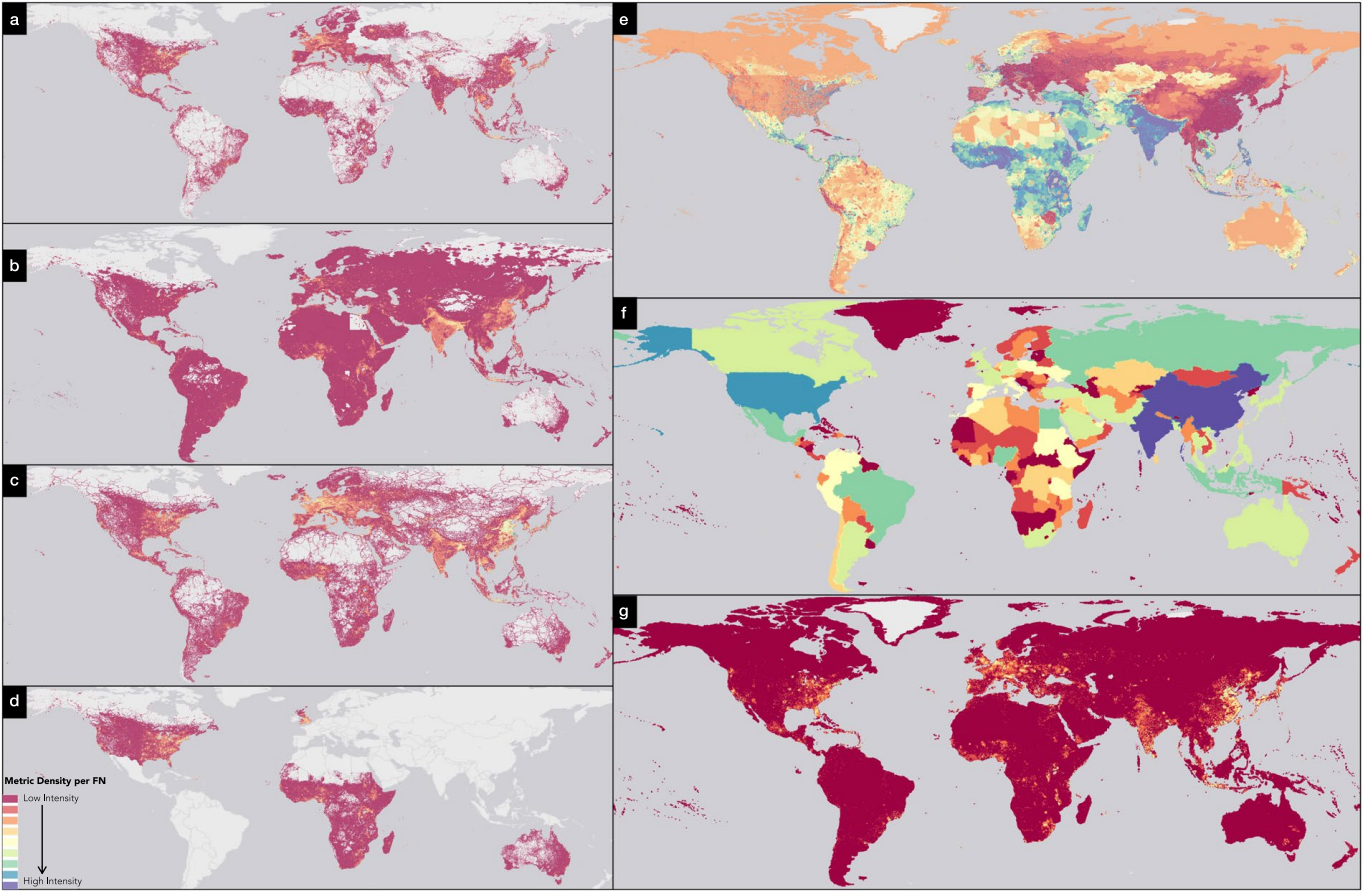
Spatial spread of the base year and SSP2-derived input datasets. (**a**) Global geo-mapped roads extracted from Open Street Maps. (**b**) Global geo-mapped population count for 2020 at 100 m resolution derived from the World POP project. (**c**) Global human-made built-up areas extracted from Copernicus Land Monitoring Program GLC V3.0.1 2019. (**d**) building footprint polygons derived from big data sources for selected continents and countries. For panels (**a**–**d**) the brighter yellow colour represents relatively high values of respective metrics in the datasets, with gradation to red colour representing low values of respective metrics in the datasets. The presence of a light grey colour represents the absence of data in the respective datasets with dark grey representing the ocean. (**e**) global change in geo-mapped population for SSP2 narrative. Red-coloured areas have the relatively lowest growth in population between 2020 and 2050, with blue-coloured areas representing the relatively highest growth in population. (**f**) country-wise change in GDP for SSP2 narrative. Red-coloured areas have the relatively lowest growth in GDP between 2020 and 2050, with blue-coloured areas representing relatively high GDP growth. (**g**) Global change in geo-mapped built-up areas for SSP2 narrative. Red-coloured areas have a relatively lowest change in the Built-up area between 2020 and 2050, with yellow-coloured areas representing the relatively highest change in the Built-up area.

Next, we chose 2020 as our base year with 2030, 2040, and 2050 as our medium-term time horizon projection years. Primary datasets collected during this study can be categorised into either a vector dataset - big data derived base year building footprint polygons (BF20), Open Street Maps (OSM)^31^ derived base year building footprint (BF20_OSM) and global geo-mapped base year roads (RL20) or raster datasets - base year global population count (PPLN20), base year global built-up extent (BU20), future SSP derived griddled population (PPLN_X,Y_), future SSP derived griddled built-up extent (BU_X,Y_), and future country wise SSP derived GDP (GDP_X,Y_), where X is the SSP narrative and Y is the year. The attributes of the different base year and SSP-derived datasets are documented in Table 1 with a visual depiction in Fig. 2.

**Table 1:** Base year layers used in this study along with their attributes.

Type	Layer	Type	Region	Attribute	Format	Size
Base Year	FN	Fishnet Grid	Global	~3.5 million polygons	Vector Polygon	N.A.
Base Year	BF20	2020 Building Footprint	USA, Canada, UK, Australia, Africa	~700 million buildings	Vector Polygon	~100 GB
Base Year	BF20_OSM	2020 Building Footprint	Rest of the world - OSM	~250 million buildings	Vector Polygon	~200 GB
Base Year	PPLN20	2020 Population Count	Global	100 m Resolution	Raster	~1GB
Base Year	BU20	2020 Built-up Area	Global	100 m Resolution	Raster	~3GB
Base Year	RL20	Road Length	Global	~34 million km	Vector Polylines	~100 GB
Future	PPLNX,Y*	SSP derived population count	Global	1/8 degree	Raster	N.A.
Future	BUX,Y*	SSP derived built-up extent	Global	1/8 degree	Raster	N.A.
Future	GDPX,Y*	SSP derived country-wise GDP	Global	Country wise	Vector Polygon	N.A.

*where “X” is the SSP narrative number, “Y” is the year for which the respective metric is provided.

The building footprint data collected from the big data sources (BF20), had full country coverage for base year building polygon data in the USA, UK, Australia, and Canada. Full continental coverage was available for Africa except for the North African region including countries above the Sahara Desert. For the rest of the world, building polygon data was derived from Open Street Maps, but the spatial coverage was sporadic with good spatial coverage only available for the European continent. This mismatch between the completeness of OSM-derived building footprints (BF20_OSM) encouraged us to create our own OSM Gap Detection application to capture selected data that has full completeness based on our FN grid (Usage Notes). The base year population count data (PPLN20) covers the entire global landmass hence no further filtering or sampling of the dataset was required.

The base year global built-up extent dataset (BU20) had global coverage for the year 2019. The built-up layer captures the extent of human-made modifications on the earth. Using a suite of remote sensing techniques, these structures can be isolated from the natural landscape and the area occupied by these structures can be converted into a raster grid where each grid cell can represent either the built-up area contained within it or the percentage of area that is built-up. Naturally, built-up extent will capture roads, carparks, industrial sites, airport runways etc. that do not form part of the building footprint and can sometimes cover 2–3 times more area than a building footprint in a built-up raster cell^23^. To account for this, we created an ML model to downscale the built-up extent to the estimated rooftop area which we will discuss in the Machine Learning model section.

**Fig. 3:**
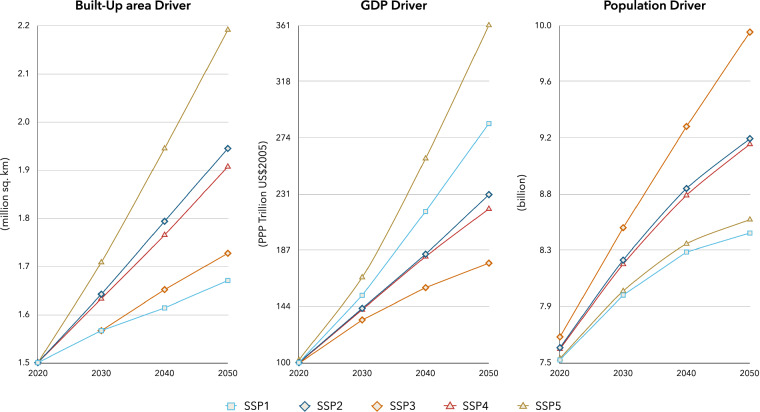
Global trend in the SSP-derived drivers.

The next step in our study after collection of base datasets for the year 2020 was to collect SSP-derived datasets for the years 2020, 2030, 2040 and 2050. In total, we collected SSP-derived data for gridded population, built-up extent, and GDP per country data for the years 2020–2050 (Fig. 3). The gridded population count dataset and built-up extent dataset were available as raster datasets at 1/8-degree spatial resolution, with the GDP per country dataset being mapped to respective country boundaries using an administrative boundary dataset from GADM project V3.6 (https://gadm.org/data.html).

### Base year calibration and spatial harmonisation

After the collection and verification of base year datasets and SSP-derived datasets, we conducted a harmonisation of the base year across the datasets. This base year harmonisation was conducted for BU20 and BF20 layers. We assumed that the 2019 built-up extent of our BU20 layer represented the 2020 data points. Similarly, the BF20 layer polygon which contains building footprint information from multiple years across different datasets was assumed to represent building footprints for the year 2020. These assumptions add a component of uncertainty in the harmonisation as some buildings constructed during the year 2020 are not part of the training dataset, but at a global scale, these assumptions will have minimal effect on the final output of the study due to the design of our ML framework.

#### Base year data aggregation

After temporally harmonising the datasets to a common base year, we aligned the datasets on a common spatial resolution and projected coordinate system. For this, we mapped the base year datasets to the FN grid. We overlayed the FN grid on top of the BF20, PPLN20, BU20 and RL20 datasets and used a cookie-cutter approach to cut and aggregate the datasets within each unique FN grid cell. Next, the BU20 layer boundary inside each FN was chosen as the region of interest and any data point outside this BU20 boundary but inside the FN boundary was not considered. This provided us with the first stage of spatial harmonisation where only datapoints inside the BU20 layer extents were considered. To achieve this, we used the area outside the BU20 layer as a masking layer to select data points that are not masked.

**Fig. 4:**
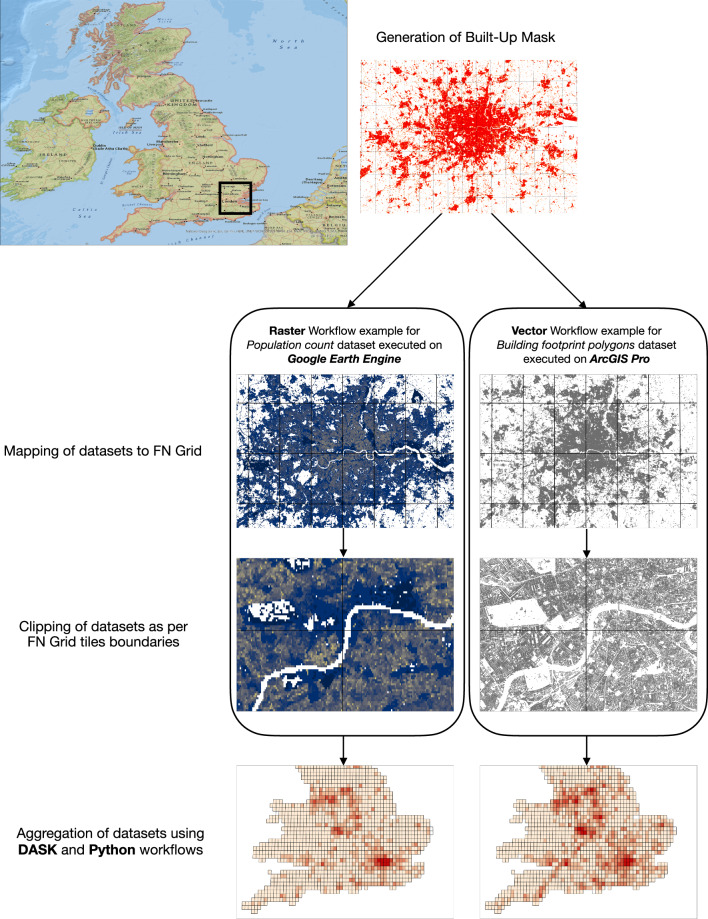
Process flow of data aggregation for FN grid. Visualisation of the workflow for UK with zoomed in view for London. The process starts with the creation of an FN grid of 1/8 degree resolution over global land mass. Next, the built-up extent layer was used as a masking layer to delineate areas where built-up structures are present in the year 2020. The masking layer along with the FN grid is then used to map vector and raster datasets to the FN grid that underlies the masking layer. Finally, the vector and raster dataset values are aggregated for each fishnet to generate a single value per FN grid cell. Here the vector datasets intersecting the FN boundary are split at the boundary and are aggregated to the respective FN grid cells while the raster datasets are aggregated using a weighted sum. Vector dataset processing is done on ArcGIS PRO, Raster dataset processing on Google Earth Engine and post-processing in python based DASK^49^ module.

The base year vector datasets representing non-masked BF20 and RL20 datasets were processed on the ArcGIS PRO V2.8 platform, where we used the inbuilt multicore processing enhancements to process the cutting and aggregation of vector datasets at scale. After the cutting step, each building polygon and road polyline feature inside each unique FN grid cell was aggregated to represent a single value per FN grid cell. It should be noted that a polygon falling on the FN grid cell boundary was intersected at the boundary and only the area of the polygon inside of the respective FN was attributed to that FN, Fig. 4.

The base year raster datasets representing non-masked PPLN20 and BU20 datasets were processed on the Google Earth Engine platform^32^. Both the datasets were clipped at the boundary of the overlapping FN and the pixels completely inside the FN were aggregated as is, with pixels falling on the boundary being aggregated using weighted summation. Here, the value attribution of the pixel in consideration was calculated based on the area of the pixel inside the FN. It should be noted that while the PPLN20 dataset represents a simple population count at 100 m resolution, the BU20 layer pixel represents the percentage of built-up area inside each 100 m pixel. Hence, the aggregation of BU20 pixel was undertaken by multiplying the pixel area by pixel value to represent the true built-up area represented by each 100 m resolution pixel.

#### SSP-derived data aggregation

**Fig. 5:**
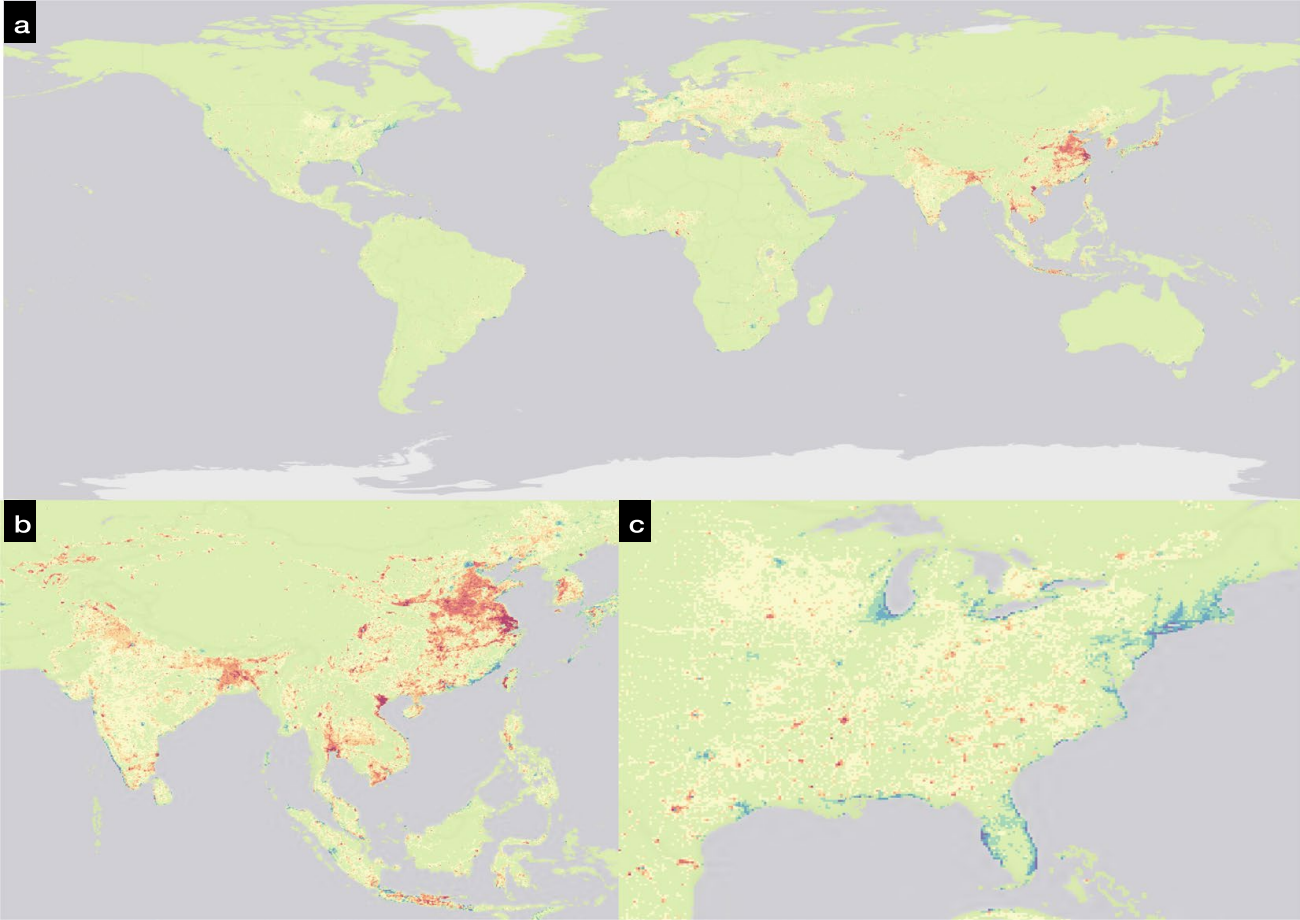
Discrepancies between BU20 layer and SSP-derived BU_X,2020_ layer. (**a**) Global FN grid cell depicting the discrepancies between BU20 and BU_X,2020_ layer. Red and orange coloured region FN grid cells have BU20 values more than BU_X,2020_ dataset values while blue FN grid cells have BU_X,2020_ values more than BU20 values. In general, the blue-coloured FN grid cells signify an overrepresentation of built-up area in BU_X,2020_ layer and red-coloured regions signify an underrepresentation of built-up area. (**b**) zoomed in the region of Asia where red-coloured FN grid cells are observed in East China with blue-coloured grid cells being observed in coastal regions. (**c**) zoomed in on the region of the east coast of the USA where blue colour FN grid cells are observed in coastal regions.

The SSP-derived population PPLN_X,Y_ and BU_X,Y_ for Y equal to 2020 were spatially harmonised to the FN grid by mapping the values from spatially harmonised PPLN20 and BU20 datasets derived in the previous steps. This aids in first providing a common base year value for estimation of future aggregated rooftop areas per FN grid cell and second removes any mismatch of data points and data values between the base datasets and SSP-derived datasets. The mismatch between the data points occurred due to PPLN_X,2020_ and BU_X,2020_ using exogenous methodologies and frameworks to estimate the values in their respective datasets. As an example, the BU_X,2020_ dataset points depicting the presence of built-up area was derived from a model that uses the GHSL^33^ layer from JRC for the year 2015 thereby not incorporating some newly developed areas in east China (Fig. 5). Additionally, the mismatch between data values can occur when for an FN grid cell BU_X,2020_ layer either under or over-represents the value depicted by the BU20 dataset. As a result of these mismatches, for a BU20 layer’s global aggregated built-up area of 1.46 million km^2^, the BU_X,2020_ layer only represents 0.98 million km^2^ of global aggregated built-up area. This highlights the importance of harmonising the datasets both at a common temporal and spatial scale.

After harmonising the PPLN_X,2020_ and BU_X,2020_ datasets for each of the SSP scenarios, the future datapoint and data values per FN grid cell of the respective datasets were recalculated using the following:

3.1 $$PPL{N}_{X,Y}=\left(PPL{N}_{X,Y}^{* }-PPL{N}_{X,2020}\right)+PPLN20$$

3.2 $$B{U}_{X,Y}=\left(B{U}_{X,Y}^{* }-B{U}_{X,2020}\right)+BU20$$

where, for each unique FN grid cell, X is the SSP scenario, Y is the year for which datapoint and value are calculated, PPLN20 is the base year population count and BU20 is the base year built-up area. The (*) nomenclature depicts future metrics before recalculation. This effectively captures the absolute growth in the metrics per FN grid cell over the harmonised base datasets. For GDP value per FN grid cell, we devised population-weighted down mapping of country-level GDP value using the following:

3.3 $$GD{P}_{X,Y}=\frac{GD{P}_{C,X,Y}}{PPL{N}_{C,X,Y}}* PPL{N}_{X,Y}$$

where, for each unique FN grid cell, X is the SSP scenario, Y is the year for which datapoint and value are calculated, and C is the country for which aggregated metrics are calculated at the country level. This GDP downscaling methodology creates a new feature layer representing GDP-weighted population count per FN grid cell for training our ML model discussed in the next section. Finally, we create the population density layers for both base year datasets and SSP-derived datasets using the following.

3.4 $$PD20=\frac{PPLN20}{F{N}_{Area}}$$

3.5 $$PPLN{D}_{X,Y}=\frac{PPL{N}_{X,Y}}{F{N}_{Area}}$$

where, for each unique FN grid cell, X is the SSP scenario, Y is the year for which the datapoint and data value are calculated and FN_Area_ is the geodesic area occupied by the FN grid cell.

### Machine learning model

**Fig. 6:**
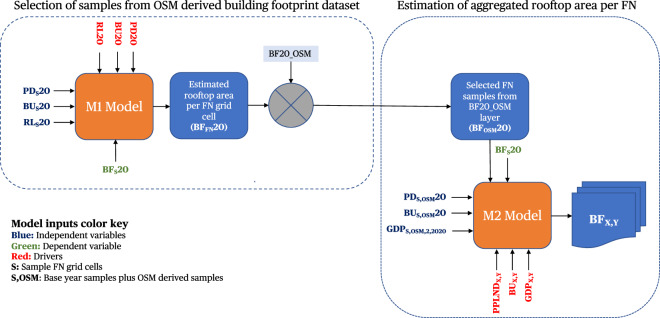
Overview of ML framework. The ML framework was divided into two stacked XGBoost models. The first model “M1” aided in the selection of samples from the global OSM building footprint dataset (BF_OSM_20). The second model “M2” combined the samples from the first model with BF_S_20 samples and used the SSP-derived drivers to estimate the aggregated rooftop area per FN grid cell. The first stage of the framework named “Model M1” accepted global built-up area (BU20), global road length (RL20) and global population density (PD20) as drivers to estimate global rooftop area per FN grid cell (B_FN_20) for the year 2020. The second stage of the framework named “Model M2” accepted SSP-derived global built-up area (BU_X,Y_), global downscaled GDP (GDP_X,Y_) and global population density (PPLND_X,Y_) to estimate global rooftop area per FN grid cell (B_X,Y_) where X is the SSP narrative and Y is the estimation year. Overall, the framework records an error of ± 0.1 km^2^ per 1/8-degree FN tile when predicting the dataset used to train the model.

We designed a ML-based framework based on XGBoost ML model^34^ to estimate aggregated rooftop area per FN grid cell. The ML framework accomplishes the task of first extracting the FN grid cell from the BF20_OSM layer derived from the OSM global building footprint dataset that has complete building footprint polygon mapping and second estimating the aggregated rooftop area per sample FN grid cells. The flow of data and steps involved in the development of the ML framework are shown in Fig. 6.

#### Training M1 model

We start the development of the ML framework by extracting sample FN grid cells from the base year datasets. The FN grid cells that have complete coverage for PD20, BU20, RL20 and BF20 datasets are selected as sample FN grid cells and the extracted sample layers are named here as PD_S_20, BU_S_20, RL_S_20 and BF_S_20 respectively. The PD_S_20, BU_S_20, and RL_S_20 sample FN grid cells then act as independent variables with BF_S_20 acting as the dependent variable for the M1 model. The M1 model is then trained by using a 10-fold cross-validation strategy and 1000 hyper-tuning iterations. The 10-fold cross-validation strategy enables the use of a complete input dataset for training purposes and aids in reducing the problem of overfitting in conjunction with 1000 rounds of hyper-tuning iterations. The trained M1 model then accepts PD20, BU20, and RL20 layers as drivers to estimate the aggregated gross rooftop area for all the global FN grid cells, BF_FN_20 layer.

#### Extraction of OSM samples

At this stage, we have a global estimate of rooftop area for the year 2020 which we then use to extract samples from the BF20_OSM layer. For this, we compare at the FN level the values of BF_FN_20 and BF20_OSM layer. For the FN grid cells where the ratio between BF20_OSM and BF_FN_20 is between 1.1 and 0.9 i.e., where BF20_OSM values show 90–110% of BF_FN_20 values, those FN grid cells are selected for their completeness of building footprint mapping and extracted as BF_OSM_20 sample layer. This comparison between M1 model predicted values and OSM-derived values also lends itself to the development of an OSM Gap detection tool which we discuss further in Usage Notes.

#### Training M2 model

**Fig. 7:**
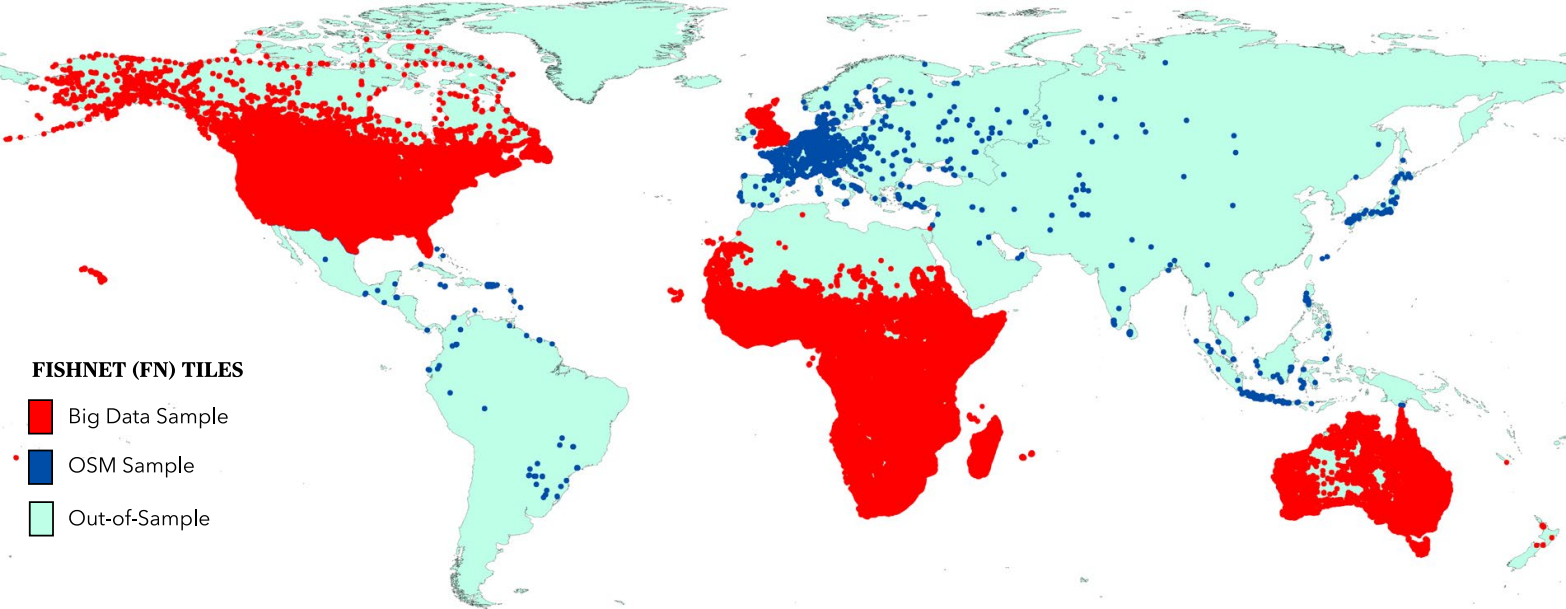
Global distribution of sample FN grid cells. The spatial spread of sample FN tiles used in our analysis amounted to 148,441 FNs for big data-derived samples and 2,654 FNs for OSM-derived samples. For FN grid cells covering the USA, Canada, Africa, UK, and Australia BF20 layer was used. For the rest of the world, OSM-derived FN grid cell was used after selecting them from inferencing the M1 model.

After tuning, training, and inferencing of BF_OSM_20 layer from the M1 model, we shift our focus to the M2 Model which will enable the estimation of global gross aggregated rooftop area per FN grid cell for SSP narratives. For this, we combine the BF_S_20 samples from the base year dataset with BF_OSM_20 samples. We also resample PD20, BU20 and GDP_X,Y_ layers to collect samples based on FN grid cells covering our combined building footprint samples to generate PD_S,OSM_20, BU _S,OSM_20 and GDP_S,OSM,2,2020_ layers. The GDP_S,OSM,2,2020_ layer here represents population-based downscaled GDP per sample FN grid cell for samples covering base year and OSM-derived Building footprint FN grid cells for SSP2 narrative and 2020 year. The PD_S,OSM_20, BU_S,OSM_20, GDP_S,OSM,2,2020_ sample FN grid cells then act as independent variables with BF_S_20 and BF_OSM_20 acting as dependent variables for the M2 model. The final sample FN grid cells used in our study are shown in Fig. 7 with building footprint attributes recorded in Table 2.

**Table 2:** Attribute of building footprint samples used for model training.

Layer	Sample Areas	Input Rooftop Area (km^2^)	# Individual Polygons
BFs20	Australia	2,418	~10 million
BFs20	UK	3,450	~33 million
BFs20	USA	29,930	~144 million
BFs20	Canada	2,500	~19 million
BFs20	Africa	17,166	~300 million
BF_OSM_20	OSM	21,000	~140 million

**Fig. 8:**
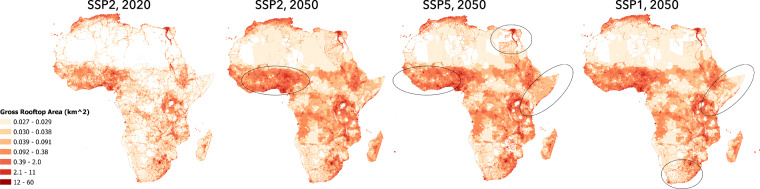
Output for BF_X,Y_ Layer for African Continent for selected SSPs and times-steps. Illustration of outputs of M2 model derived assessment of rooftop area per FN for African continent for 2020 base year and 2050 future year for SSP2, SSP1 and SSP5 narrative. The black circles highlight selected regions where growth dynamics can be observed across selected SSPs based on the 2020 year.

The M2 model is trained by using a 10-fold cross-validation strategy and 1000 hyper-tuning iterations. At the conclusion of this step, we have our final M2 model which then accepts PPLND_X,Y_, BU_X,Y_ and GDP_X,Y_ layers as drivers to estimate a global BF_X,Y_ layer for five SSP narratives and years ranging from 2020–2050. The final BF_X,Y_ layer is stored as GeoPackage files having 1/8 degree FN grid cell resolution with a value representing the aggregated gross rooftop area inside the FN grid cell for further analysis, Fig. 8.

Although the trained M1 model in conjunction with SSP-derived drivers can aid in the generation of the final BF_X,Y_ layer, we could not implement this as RL20 layer data is only available for the base year of 2020 and multivariate regression would be required to estimate its value beyond 2020 which would add an extra layer of uncertainty in our results. Additionally, the selection of BU_S,OSM_20 and the merger of this layer with BF20 layer provided us with additional global data points to retrain a new model M2 which would be more compliant with global trends rather than just the countries/regions covered by BF20 dataset.

## Data Records

**Fig. 9:**
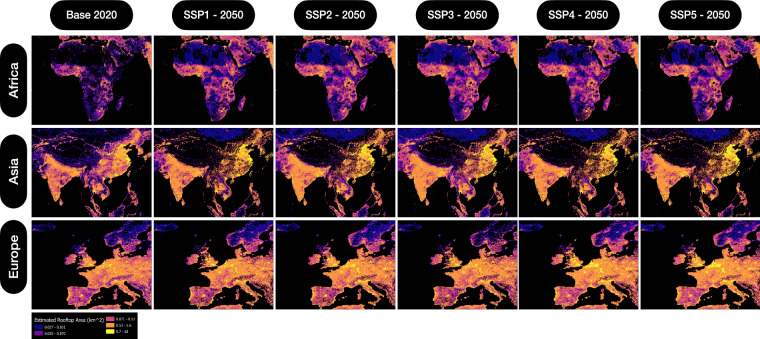
Visual depiction of BF_X,Y_ layer for selected global regions. The image panels depict the pixel-wise output of BF_X,Y_ layer classified by a graduated colour ramp. Each pixel in the panel represents the aggregated gross rooftop area per FN grid cell. Growth in rooftop area per FN grid cell can be observed for East China, West Africa, and Central European areas.

The high-resolution datasets generated in this study contains 3,216,960 individual Fishnet tiles with 1/8 degree spatial resolution, spanning the entire globe. The main datasets along with additional files are hosted and referenced on Zenodo^35^ (10.5281/zenodo.11085013). The dataset covers all countries except Antarctica. Selected regional outputs of the study are shown in Fig. 9. To enable easy integration in the workflows, we have provided the main datasets in the following formats:

1. ***Vector dataset:*** The global gross estimated rooftop area per FN grid cell for each SSP narrative is provided as a *Geopackage (.gpkg)* file (*Results_Vis.gpkg*) with polygon geometries at 1/8-degree spatial resolution in an EPSG:4326 coordinate system. The *attribute table* of this file contains *FN_ID* column representing the FN grid cell ID, and other columns representing the FN_ID specific assessed rooftop area. The assessed gross rooftop area columns are sequenced as *BF_X_Y* with *X* having values as *1, 2, 3, 4, and 5* for *SSP1, SSP2, SSP3, SSP4, SSP5* narratives with *Y* representing the assessment year having values as *20, 30, 40, and 50* for years *2020, 2030, 2040, and 2050* and with *km*^2^ units. In addition, a CF column is added for each FN_ID entry that documents the Capacity Factor for rooftop solar PV based on the World Bank solar atlas^36^.
2. ***Raster datasets:*** The global gross estimated rooftop area per FN grid cell for each SSP narrative is provided as a *geotiff (.tif)* files with LZW compression in an EPSG:4326 coordinate system. The assessed gross rooftop area datasets are sequenced as *BF_X_Y* with *X* having values as *1, 2, 3, 4, and 5* for *SSP1, SSP2, SSP3, SSP4, SSP5* narratives with *Y* representing the assessment year having values as *20, 30, 40, and 50* for years *2020, 2030, 2040, and 2050* and with *km*^2^ units.
3. ***Numerical dataset:*** The global gross estimated rooftop area per FN grid cell for each SSP narrative is provided as a *parquet (.parquet)* file *(Results.parquet)*. This file contains *FN_ID* column representing the FN grid cell ID, and other columns representing the FN_ID specific assessed rooftop area. The assessed gross rooftop area columns are sequenced as *BF_X_Y* with *X* having values as *1, 2, 3, 4, and 5* for *SSP1, SSP2, SSP3, SSP4, SSP5* narratives with *Y* representing the assessment year having values as *20, 30, 40, and 50* for years *2020, 2030, 2040, and 2050* and with *km*^2^ units. In addition, a *CF* column is added for each FN_ID entry that documents the Capacity Factor for rooftop solar PV based on the World Bank solar atlas.

In addition to the main datasets, we have provided additional files to enable generating the vector and numerical datasets from this study:

1. ***M2_Model.json:*** This file contains the frozen parameters of the M2 model in*.json* format generated from XGBoost version 2.0.3
2. ***SSP_drivers.parquet:*** This file contains the driver data used for generating the main dataset in our study
3. ***FN_MAP.parquet:*** This file contains the boundary information for each fishnet grid tile in a Well Known Text *(WKT)* format.
4. ***Prediction.ipynb:*** This file provides a python notebook interface to generate inferencing from *M2_Model.json* using *SSP_drivers.parquet* file. In addition, this file also generates the numerical dataset and converts it into vector dataset using *FN_MAP.parquet* file.
5. ***environment.yaml:*** This file contains the frozen configuration of python virtual environment used to generate the results presented in this study.

## Technical Validation

### Input validation

The datasets presented in this study have undergone end-to-end technical validation for the base year of 2020. The validation is performed for M1 and M2 model inputs, the performance of M1 and M2 models, the validity of outputs of M1 and M2 models and finally verification of estimations generated by the M2 model. For datasets covering the years 2030–2050, we could not provide a true verification of data validity as they represent the future, but the high accuracy of 2020 data suggests strong model veracity which provides high confidence in these outputs. The input validation of the base year datasets and SSP-derived drivers are presented in Table 3 as a link to the validation reports generated by either the data providers or the peer-reviewed publication which form the basis of the data. Due to the scale of the dataset, assumptions and the limitation of methods used, the big datasets used in this study are expected to have errors at a higher resolution when verifying at a per building level, but at an aggregated country/ regional spatial resolution these datasets have shown acceptable performance.

**Table 3:** Input data validation.

Dataset	Format	Validation study link
Building Footprints	Vector	Heris, M.P., Foks, N.L., Bagstad, K.J. *et al*. A rasterized building footprint dataset for the United States. Sci Data 7, 207 (2020).
		10.1038/s41597-020-0542-3
		W. Sirko, S. Kashubin, M. Ritter, A. Annkah, Y.S.E. Bouchareb, Y. Dauphin, D. Keysers, M. Neumann, M. Cisse, J.A. Quinn. Continental-scale building detection from high resolution satellite imagery.
		arXiv:2107.12283, 2021
Population	Raster	Lloyd, C., Sorichetta, A. & Tatem, A. High resolution global gridded data for use in population studies. Sci Data 4, 170001 (2017).
		10.1038/sdata.2017.1
Road	Vector	Barrington-Leigh, C., & Millard-Ball, A. (2017). The world’s user-generated road map is more than 80% complete. PloS one, 12(8), e0180698.
		10.1371/journal.pone.0180698
Built-up area 2020	Raster	Tsendbazar, N.E., Tarko, A., Linlin, *et al*. (2020): Copernicus Global Land Service: Land Cover 100 m: Version 3 Globe 2015–2019: Validation Report; Zenodo, Geneve, Switzerland, September 2020;
		10.5281/zenodo.3938974
SSP derived Built-up area 2020–2050	Raster	Gao, J., O’Neill, B.C. Mapping global urban land for the 21st century with data-driven simulations and Shared Socioeconomic Pathways. Nat Commun 11, 2302 (2020).
		10.1038/s41467-020-15788-7
SSP derived Population 2020–2050	Raster	KC, S. & Lutz, W. The human core of the shared socioeconomic pathways: Population scenarios by age, sex and level of education for all countries to 2100. Global Environmental Change vol. 42 181–192 (2017).
		10.1016/j.gloenvcha.2014.06.004
SSP derived GDP 2020–2050	Dataset	Dellink, R., Chateau, J., Lanzi, E. & Magné, B. Long-term economic growth projections in the Shared Socioeconomic Pathways. Global Environmental Change vol. 42 200–214 (2017).
		10.1016/j.gloenvcha.2015.06.004

### Model validation on sample FN tiles

The learning accuracy of the M1 and M2 models is determined by the significance of the correlation between the dependent and independent variables used to train the model. Further, a 10-fold cross-validation strategy to expose the models to various combinations of input data to reduce model overfitting was used. Additionally, the distribution of model output with respect to the dependent variables and the spread of the errors were evaluated to choose the best model. It was observed that the M2 model has a slight tendency to underestimate ground truth.

**Fig. 10:**
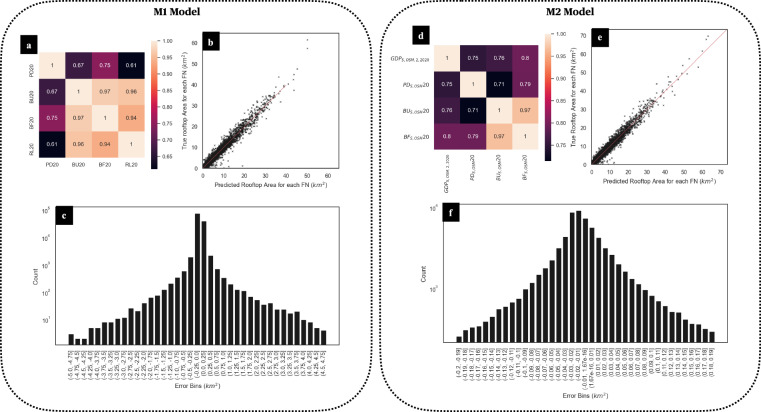
Performance metrics of M1 and M2 models on sample FNs. (**a**) Correlation heatmap representing pearson’s correlation between pairs of independent and dependent variables of the M1 model. High correlation can be observed for the dependent variable (BF20) and independent variables (PD20, RL20, and BU20). (**b**) a graph representing the relationship between the M1 model’s dependent variable and predicted values. High fidelity can be observed between a dependent variable and predicted values. (**c**) the spread of difference between dependent variable value and predicted value from the M1 model at a per FN grid cell basis. The majority error concentration is around ± 0.1 km^2^ for a 1/8 degree FN grid cell. (**d**) Correlation heatmap representing Pearson’s correlation between pairs of independent and dependent variables of the M2 model. High correlation can be observed for the dependent variable (BF_S,OSM_20) and independent variables (PD_S,OSM_20, GDP_S,OSM,2,2020_20, and BU_S,OSM_20). (**e**) a graph representing the relationship between the M2 model’s dependent variable and predicted values. High fidelity can be observed between the dependent variable and predicted values. (**f**) spread of difference between dependent variable value and predicted value from M2 model at a per FN grid cell basis. The majority error concentration is around ± 0.05 km^2^ for a 1/8 degree FN grid cell with slight left skewness in the error distribution leading to model prediction showing a slight underestimation of ground truth at the FN grid cell level.

**Fig. 11:**
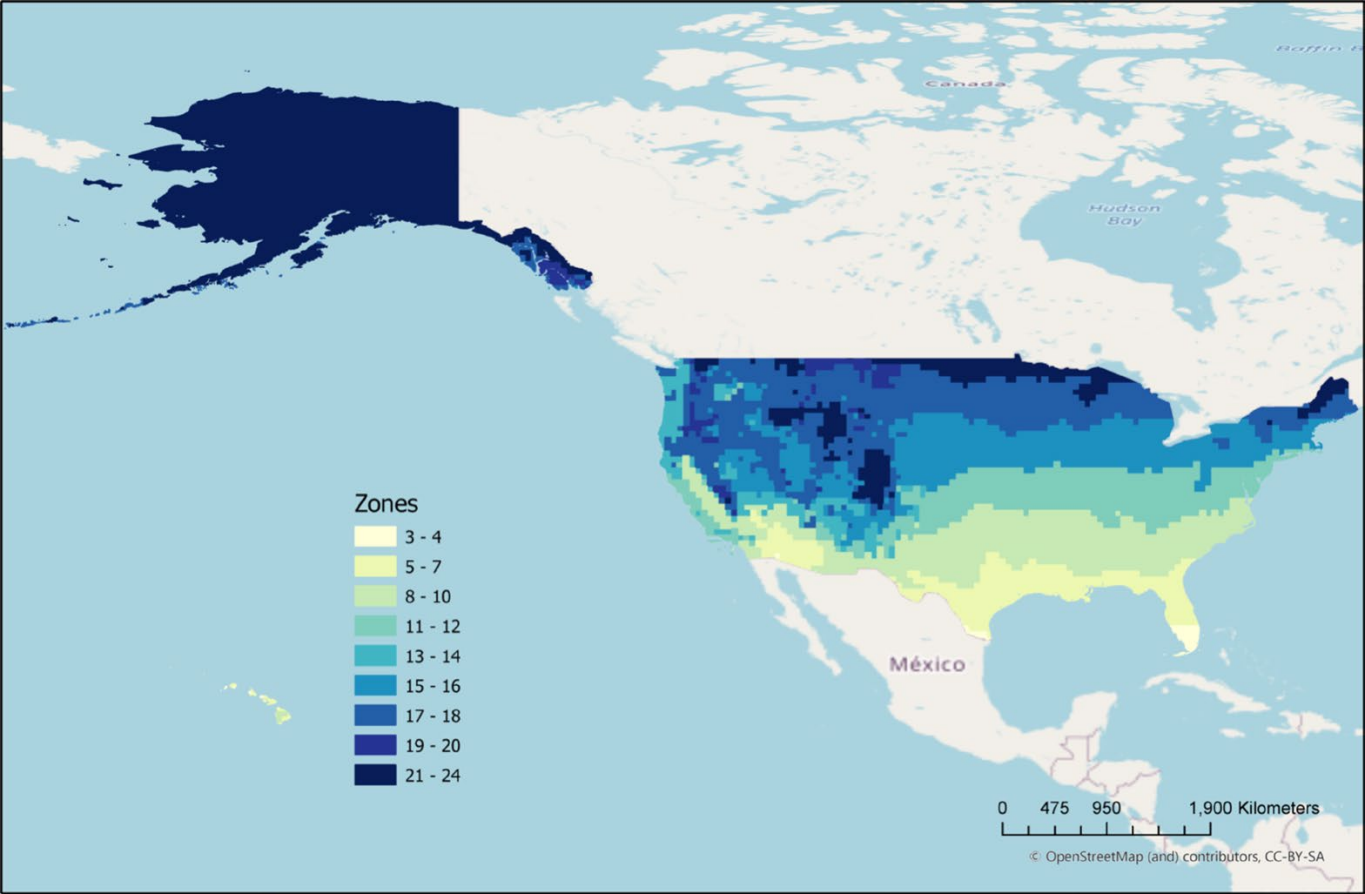
Map of climate zones over the USA.

The final output of the M2 model (BF_X,Y_) was further evaluated for discrepancies between aggregated country-wise input base year big data derived BF20 values and aggregated country-wise M2 models estimated outputs for SSP2 narrative in the year 2020 (BF_2,2020_). These evaluations were conducted by aggregating the FN grid cell values for those FN grid cells that fall within the geographic boundaries of the country being evaluated. Overall, we observed high fidelity between the ground truth and estimated values at a country level. On a higher spatial resolution, we also compared the sub-national level estimations for the USA based on ASHRAE USA Climatic regions. Here also high fidelity was observed between ground truth and predicted values. Figures 10, 11 and Table 4 document the results of these checks.

**Table 4:** Result comparison of M2 Model’s output on seen training data.

Testing Attribute	Spatial Level	Name	Ground Truth (BF20) (km^2^)	M2 Model’s output (km^2^)	Absolute percentage error (%)	Source
Seen by Model	Regional	USA Climatic Zone 3–4	734	633	13.76	Fig. 11
Seen by Model	Regional	USA Climatic Zone 5–7	76	108	42.11	Fig. 11
Seen by Model	Regional	USA Climatic Zone 8–10	4,103	3,789	7.65	Fig. 11
Seen by Model	Regional	USA Climatic Zone 11–12	701	706	0.71	Fig. 11
Seen by Model	Regional	USA Climatic Zone 13–14	5,531	5,532	0.02	Fig. 11
Seen by Model	Regional	USA Climatic Zone 15–16	1,028	1,074	4.47	Fig. 11
Seen by Model	Regional	USA Climatic Zone 17–18	1,426	1,267	11.15	Fig. 11
Seen by Model	Regional	USA Climatic Zone 19–20	5,666	5,819	2.7	Fig. 11
Seen by Model	Regional	USA Climatic Zone 21–24	216	218	0.93	Fig. 11
Seen by Model	Country Level	USA	29,681	29,447	0.79	—
Seen by Model	Country Level	UK	3,450	3,492	1.22	—
Seen by Model	Country Level	Africa	17,166	17,525	2.09	—
Seen by Model	Country Level	Australia	2,418	2,527	4.51	—
Seen by Model	Country Level	Canada	2,500	2,753	10.12	—

### Result validation on unseen datasets

**Fig. 12:**
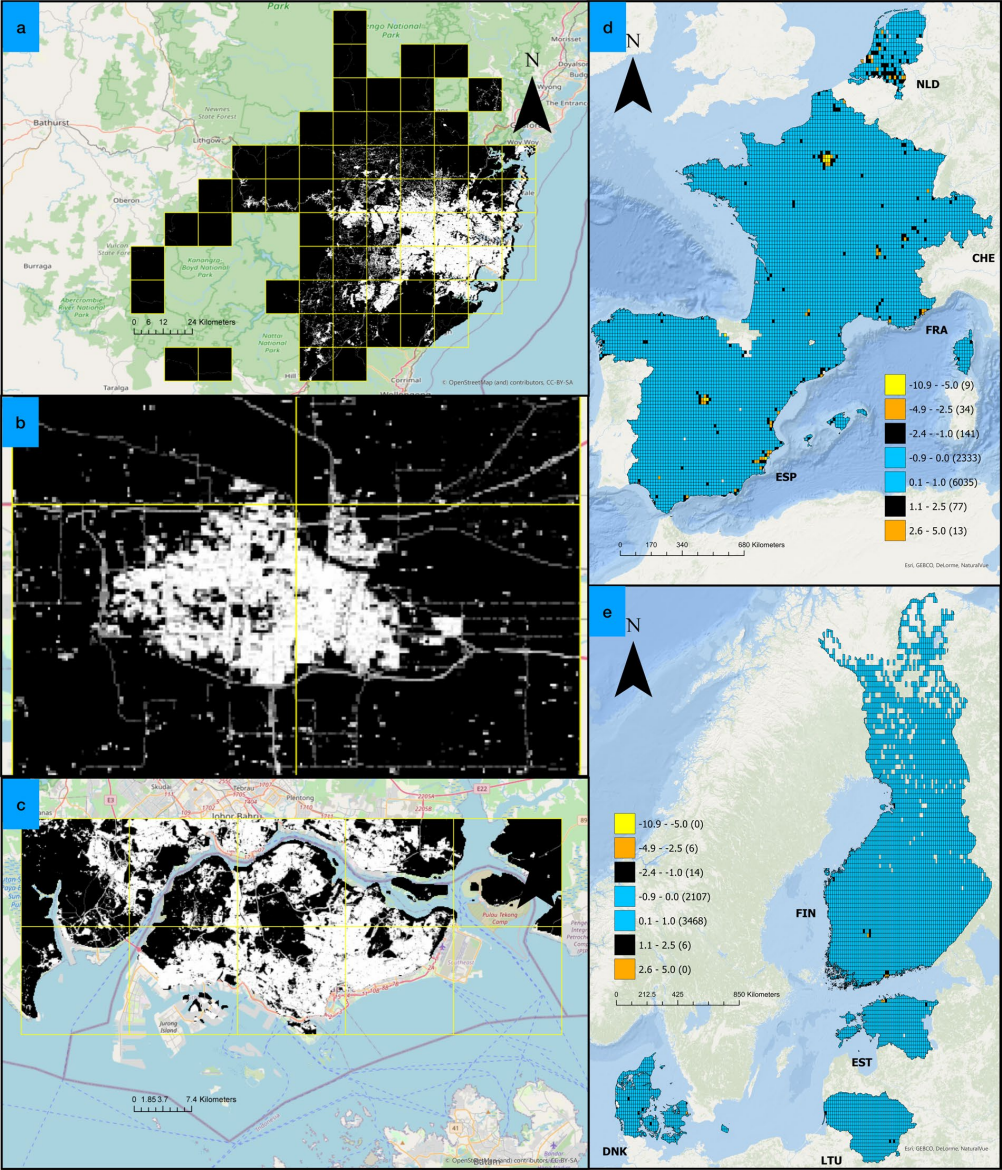
Illustration of boundaries and results from the validation of unseen data. (**a**–**c**) Boundary of test sample over greater Sydney, Kansas and Singapore ROI respectively. The FN grid is marked with yellow colour, the black area is the non-built-up area inside the FN tile and the white/grey area is the built-up area inside the FN tile. (**d,e**) Illustration of the difference between EUBUCCO v0.1 and BF_2,2020_ layers for selected countries. The legend represents the colormap for different error bins with values in brackets representing FN tiles within each bin. Negative values represent under-prediction and positive values represent over-prediction by the M2 model. Basemap- Open Street Map contributors, Built-up classification - Copernicus GLC V3.0.1 2019.

After verifying the M2 model’s output (BF_X,Y_) on seen/training data, further validations were performed on the unseen datasets. Here we compared our results (BF_2,2020_) i.e. M2 model’s output for SSP2 and year 2020 with EUBUCCO v0.1^4,37^ dataset for selected countries that had full data availability in EUBUCCO v0.1 dataset. The countries are Spain, France, Netherlands, Denmark, Finland, Estonia, Lithuania, Slovakia, Slovenia, Switzerland, Germany, and Luxembourg. For this, we first masked the EUBUCCO v0.1 dataset with the built-up layer in 2020 (BU20) and then mapped the resulting building footprints onto the FN grid flooded by aggregation of building footprint geometry within each FN grid tile. The second set of validation at the sub-national level was performed for the cities of Kansas, Singapore, and Sydney. Overall, we found that the results of the M2 model are within expected error ranges when compared with unseen data that is not exposed to the M2 model during training. This way, we could validate our results to a high degree of certainty by comparing results at sub-national and national spatial levels. Table 5 along with Fig. 12 documents the finding of the validations performed on unseen datasets.

**Table 5:** Result comparison of M2 Model’s output on seen and unseen data.

Testing Attribute	Spatial Level	Name	Ground Truth (BF20) (km^2^)	M2 Model’s output (km^2^)	Absolute percentage error (%)	Source
Unseen by model	Country Level	France	5,332	5,326	0.11	Fig. 12d
Unseen by model	Country Level	Spain	2,538	2,552	0.57	Fig. 12d
Unseen by model	Country Level	Slovenia	142	137	3.57	—
Unseen by model	Country Level	Finland	480	461	3.89	Fig. 12e
Unseen by model	Country Level	Germany	5,825	5,570	4.37	—
Unseen by model	Country Level	Lithuania	238	227	4.79	Fig. 12e
Unseen by model	Country Level	Slovakia	407	387	4.91	—
Unseen by model	Country Level	Switzerland	473	496	4.99	Fig. 12d
Unseen by model	Country Level	Estonia	111	96	12.95	Fig. 12e
Unseen by model	Country Level	Netherlands	1,153	991	14.01	Fig. 12d
Unseen by model	Country Level	Denmark	649	509	21.62	Fig. 12e
Unseen by model	Sub-national	Kansas	10	9	10	Fig. 12a
Unseen by model	Sub-national	Singapore	104	116	11.54	Fig. 12b
Unseen by model	Sub-national	Sydney	313	314	0.32	Fig. 12c

## Usage Notes

### Limitations

The aggregated rooftop area dataset was generated with an assumption of one-to-one mapping between the building footprint and the rooftop area. Although some building archetypes can have a larger rooftop area than building footprint due to the presence of rooftop superstructures^14^, we have not considered this due to the scale of the analysis which looks at global region of interest rather than per building. Similarly in higher latitudes due to the slope of the rooftops, the total building rooftop area can be higher than the building footprint area. Hence, it is advised to use region-specific rooftop attribute values when using these datasets for city-level analysis. Additionally, due to the nature of the ML model used for the estimation of rooftop area, we recommend an error margin of ± 0.1 km^2^ per FN grid cell. Considering the global scope of this study, we assume medium term (2020–2050) stationarity of spatiotemporal patterns learned by M2 model which limits the future projection of gross rooftop area. To mitigate the assumption of spatiotemporal stationarity, we have incorporated five different growth pathways in the form of SSPs that act as a proxy of different urban planning paradigms, thus allowing for an integrated assessment with various other factors e.g. climate change, energy systems etc. Finally, the training data to drive M2 Model is partially biased towards developed nations with only African countries and some samples from Open Street Maps providing training data for emerging economies. This imbalance in training data has manifested itself as the slight tendency for underestimation of gross rooftop area for high-density cities and conurbations.

### Application to energy system/integrated assessment modelling

**Fig. 13:**
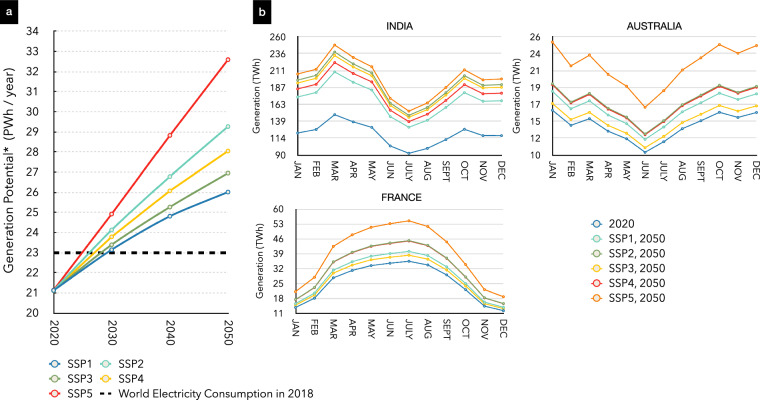
Illustration of application of this study in rooftop solar PV assessment. (**a**) Global growth in assessed rooftop solar PV potentials based on 30% rooftop availability factor and 20% panel efficiency. The values are calculated by converting the gross rooftop area to the net rooftop area using the rooftop availability factor, then the net rooftop area is converted into installed capacity and further into yearly aggregated potentials. (**b**) illustration of intra-year variability in rooftop solar PV potentials for different SSP narratives and for selected countries.

We foresee that the datasets generated in this study will be of urgent use to the energy system/Integrated assessment modelling community for assessment of rooftop Solar PV/Solar thermal technical potential^6,38,39^ applications and for building side energy systems modelling^40–42^ purposes. For energy justice^43^ and energy accessibility studies^44^, the datasets can provide invaluable information in the form of urban growth dynamics and for calibration of the building stock models. For example, in the technical potential assessment studies^45^, users can assume that rooftops are flat with solar panels being placed at the latitude-specific optimal angle. Users can also assume that the entire estimated rooftop area will be fully covered by solar panels and the panels will be devoid of shadows. This assumption culminates as our dataset representing the best-case scenario for a technical potential generation. In wider literature, a rooftop availability factor of 0.3 is used to convert gross rooftop area to net rooftop area to account for unsuitable rooftops due to orientation and slope attributes of building stocks. For the users of this dataset, we recommend using region-specific rooftop availability factors if known, else 0.3 can be used as the factor for more practical results. The net rooftop area can then directly be converted into monthly technical potentials using high-resolution solar irradiate datasets e.g. NASA MERRA 2^46^, Fig. 13.

### Application to analyse OSM spatial data completeness

Open Street Map-derived data is being used in many studies as a source of ground truth mapping and for the calibration of big data models. Additionally, raw OSM data in the form of building polygons, and road mapping is being used extensively in resource accessibility studies and vulnerability mapping^47^. A primary reason for the uptake of OSM data can be attributed to its free accessibility and the presence of more than a million active users who are updating the digital planet files on an hourly basis. Although the quantity of data that is present inside the OSM database is vast, studies using them often must do significant pre-processing to extract data that is suitable for their use case. Additionally, users of the OSM dataset struggle with the lack of validation studies done on OSM datasets.

For data attributes dealing with global roads, one study^48^ highlights that the OSM global road dataset is 80% complete. Similar studies for global building footprint datasets are currently limited to either country-level studies (https://github.com/thinkingmachines/osm-completeness) or regional studies (https://github.com/hotosm/osm-analytics). As an application of the output of our M1 model, we overlayed our predicted gross rooftop area mapped to the FN grid for the year 2020 and for the SSP2 growth narrative on top of the building footprint polygon planet dataset from OSM to estimate the completeness of the OSM dataset. To quantify the completeness, we calculated the percentage difference in the assessed gross rooftop area from our study and the calculated gross building footprint area mapped to the FN grid from OSM. The base dataset for OSM comparison was procured in August 2021.

**Fig. 14:**
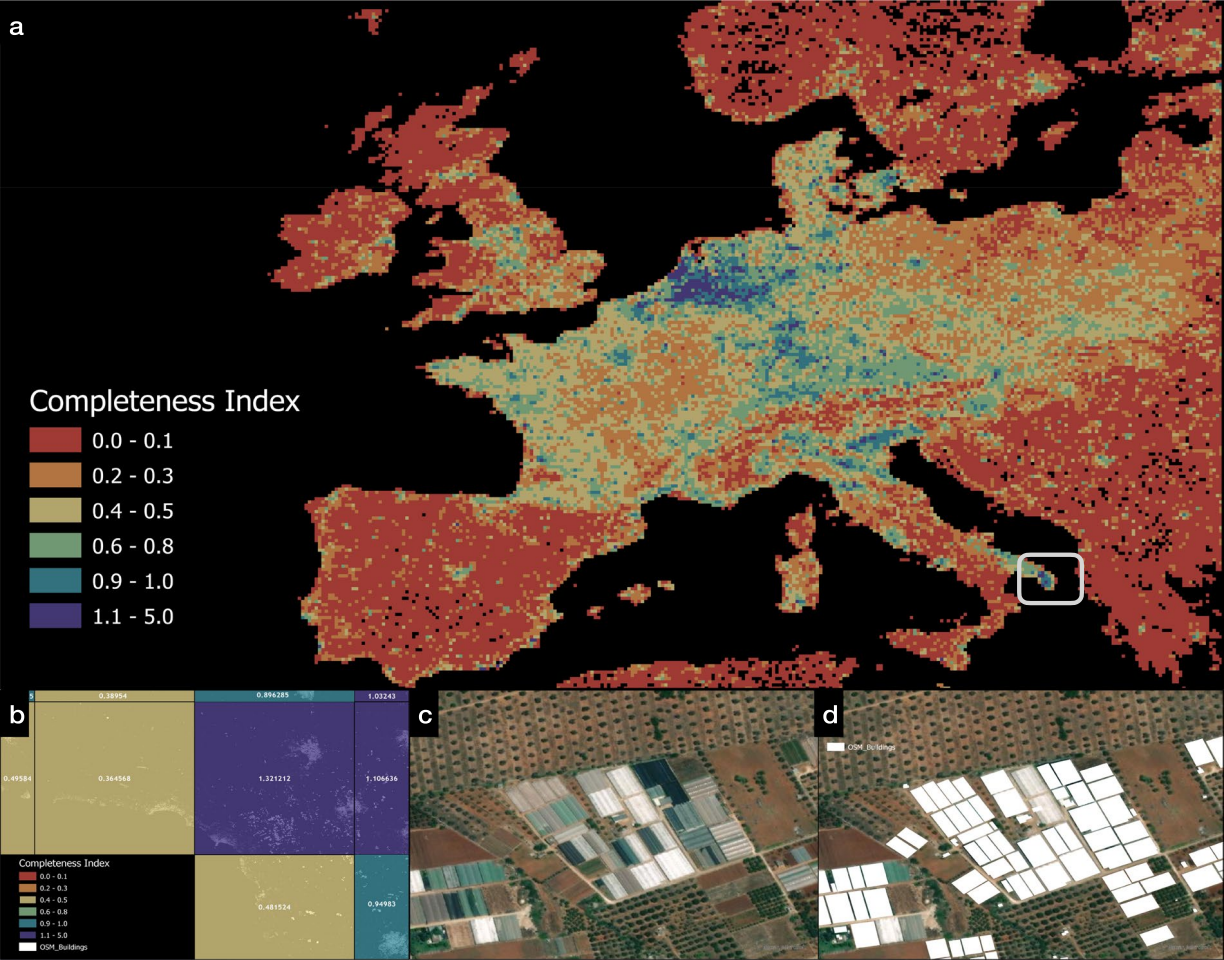
Output visualisation of OSM gap detection tool. The output of the gap detection tool dataset with each individual FN grid cell is classified by the completeness index, (**a,b**) zoomed in view of the ROI bound by the white bounding box in Fig. 14a. The values inside the FN grid cell represent the completeness index value for that FN grid cell. (**c**) image displaying a sample of an area marked inaccurately in the OSM dataset inside the FN grid cell with a completeness index of 1.32. (**d**) overlay of OSM polygon on incorrectly identified buildings in Fig. 14c where greenhouse installations have been marked as buildings leading to the FN representing value greater than 1.

In the final output of this analysis, a value of 0 represented that either OSM data is missing, or data cannot exist at that FN grid cell. A value of 1 represented that OSM dataset coverage is 100% in that FN grid cell. Any value between 0.9–1.5 was considered as representing 100% completeness of the OSM dataset as our M1 model does have under or over-prediction characteristics in some regions based on driver metrics. A value greater than 1.5 was representative of regions in OSM that may not have population presence but have OSM building polygon tags e.g., greenhouses, industrial complexes around major shipping ports etc. Since our M1 model relies on the population as an important driver, in FN grid cells having a completeness value greater than 1.5, our model gives a lower value than the OSM dataset value. Another reason for this can be attributed to the wrong tag being assigned to building polygons or the misclassification of non-building built-up structures as building polygons inside the OSM dataset. An example of a completeness value dataset is shown for Europe in Fig. 14a, with example cases of completeness value greater than 1 shown in Fig. 14b–d. A similar automated analysis can be conducted for a global dataset to quantify the completeness of the OSM dataset and direct the crowdsourced mapping of buildings to areas that are under mapped.

### Pseudocodes

#### Algorithm 1

Data Collection and pre-processing.

#### Algorithm 2

XGBoost Model Training and Estimation (Model M1).

#### Algorithm 3

Preparing training Data for Model M2.

#### Algorithm 4

XGBoost Model Training and Estimation (Model M2).

## Data Availability

We have documented within the Data Descriptor the Pseudocodes that support the methodology of this study. Codes used for inferencing results along with XGBoost model generated in this study are hosted at Zenodo (10.5281/zenodo.11085013).
